# Fatty liver index in early pregnancy predicts the risk of gestational diabetes mellitus

**DOI:** 10.3389/fendo.2026.1659379

**Published:** 2026-02-26

**Authors:** Juping Wei, Ning Ma, Liwei Bai, Qiang Lu

**Affiliations:** 1 Department of Obstetrics, First Hospital of Qinhuangdao, Hebei, Qinhuangdao, China; 2 Department of Endocrinology, First Hospital of Qinhuangdao, Hebei, Qinhuangdao, China; 3 Department of Obstetrics, Qinhuangdao Hospital for Maternal and Child Health, Hebei, Qinhuangdao, China

**Keywords:** fatty liver index, gestational diabetes, MAFLD, predictive factor, TyG

## Abstract

**Objective:**

To examine the ability of the fatty liver index (FLI) in early pregnancy to predict the risk of Gestational Diabetes Mellitus (GDM).

**Methods:**

A total of 1,004 women underwent metabolism characterization at weeks 8–12 of gestation and a 75 g oral glucose tolerance test (OGTT) at weeks 24–28 of gestation. The participants were divided into the normal glucose tolerance (NGT, n = 816) and GDM (n = 188) groups according to the OGTT results. Pregnant women were divided into three tertiles according to their FLI scores in early pregnancy. Multivariable regression analysis was performed to estimate the independent relationship between FLI and GDM.

**Results:**

The FLI values of the GDM and NGT groups were 8.47 (5.26, 13.41) and 6.10 (3.90, 10.41), the differences were significant (*P* < 0.001). The FLI values of the T1–T3 groups were 3.49 (2.74, 4.07), 6.43 (5.58, 7.51), and 14.46 (10.96, 21.58), respectively. The differences in homeostasis model assessment of insulin resistance (HOMA–IR), TyG, TG/HDL-C, LDL-C/HDL-C, TC/HDL-C, and ALT/AST between the T1–T3 groups were significant and gradually increased (*P* < 0.001). The risk of GDM in pregnant women in the highest FLI tertile was 1.881 times greater than that in the lowest FLI tertile(OR = 1.881, 95% CI: 1.049–3.374, *P* = 0.034).The cut-off point of FLI for predicting the risk of GDM was 5.108. Compared with other three indicators, FLI has better sensitivity (77.7%), but the specificity was slightly lower (41.1%).

**Conclusion:**

The early pregnancy FLI is an independent risk factor for GDM. A high FLI is predictive of GDM risk.

## Introduction

Gestational Diabetes Mellitus (GDM) refers to glucose metabolism abnormalities that occur for the first time during pregnancy and account for 80–90% of gestational hyperglycemia. GDM causes short- and long-term adverse effects on mothers and infants (1, 2) and is a major public health issue. One previous study revealed that gestational nonalcoholic fatty liver disease (NAFLD) is a major risk factor for GDM (3). Currently, metabolic dysfunction-associated fatty liver disease (MAFLD) has gradually replaced the traditional concept of NAFLD. Ludwig (4) first proposed the concept of “non-alcoholic fatty liver disease (NAFLD)” in 1980. In 2020, an international expert group from 22 countries proposed a new name, “metabolic dysfunction-related fatty liver disease (MAFLD)” (5). The definition of JMAFLD emphasizes the importance of metabolic dysfunction, including being overweight, obese, having T2DM or having at least 2 metabolic risk factors, without considering the underlying causes and comorbidities, such as alcohol consumption and viral hepatitis.

Currently, the assessment of hepatic steatosis severity primarily relies on liver biopsy or ultrasound imaging. Liver biopsy, although considered a reference standard, is invasive and associated with low patient compliance, limiting its routine use in clinical practice. Consequently, the diagnosis of metabolic dysfunction–associated fatty liver disease (MAFLD) predominantly depends on ultrasound examination. However, the diagnostic accuracy of ultrasound is constrained by operator-dependent variability and the technical specifications of the imaging equipment. The fatty liver index (FLI) is a simple and accurate marker of hepatic steatosis (6) and an effective predictor of gestational NAFLD (7). The FLI is accurate in the detection of fatty liver disease. This index can also replace ultrasound to determine the severity of hepatic steatosis. Compared with that in the normal population, the incidence of MAFLD is significantly greater in pregnant women and poses a potential risk to maternal and infant health (8).

Inflammation and insulin resistance are key pathological processes in the development and progression of MAFLD (9, 10). Chronic low-grade inflammation contributes to hepatic steatosis and fibrosis through the activation of pro-inflammatory cytokines and immune cells, while insulin resistance promotes lipid accumulation and impairs glucose metabolism in hepatocytes (11, 12). Many previous studies have shown that the FLI is a predictor of type 2 Diabetes Mellitus (13, 14). however, no studies have investigated whether the FLI is a predictor of GDM. This study examined the ability of the FLI in early pregnancy to predict the GDM risk.

## Materials and methods

### Study participants

**Figure 1:**
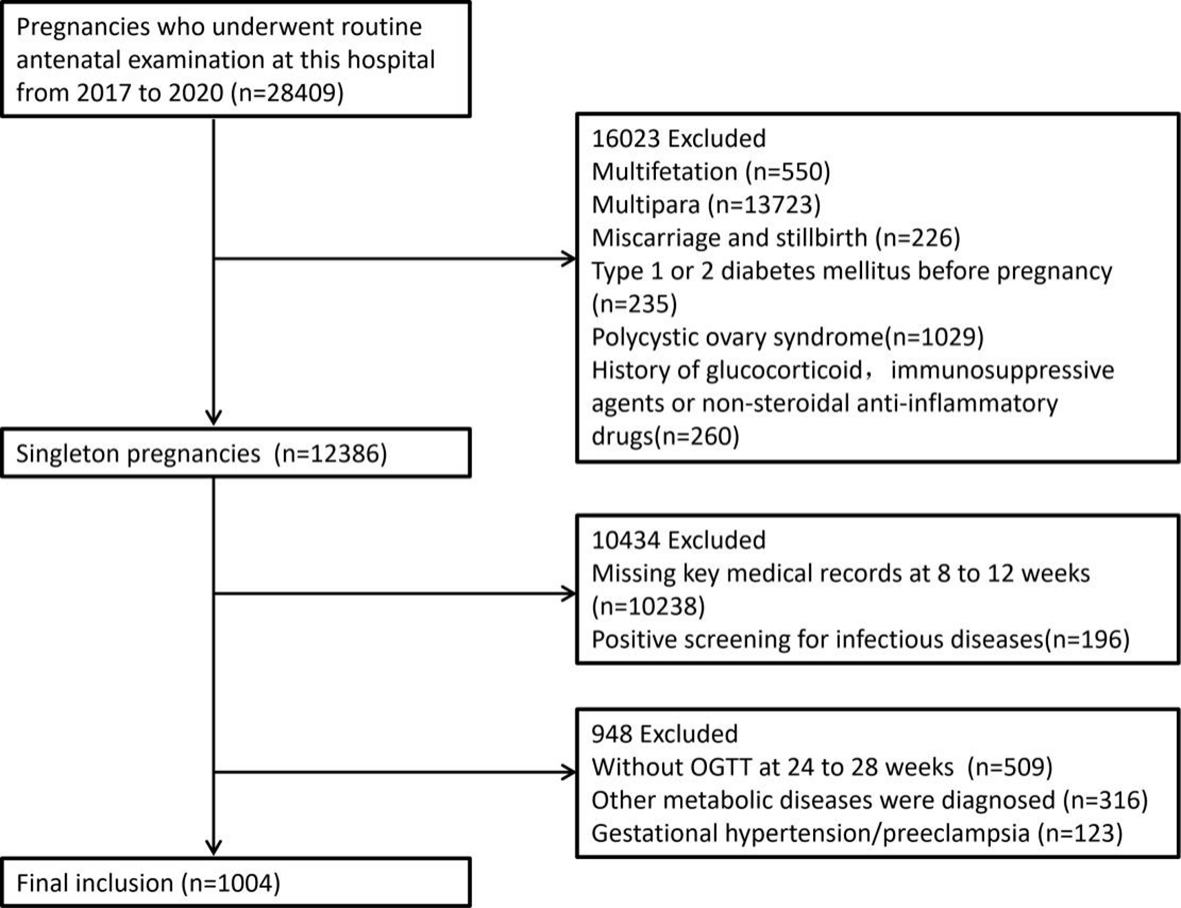
Detailed screening procedures for eligible subjects.

Pregnant women who underwent routine prenatal examinations at the Maternal and Child Health Hospital of Qinhuangdao from September 2017 to September 2020 were selected. The ages of the participants ranged from 17 to 43 years, and the mean age was 28.19 ± 3.70 years. A total of 1,004 participants were included in the baseline survey. **Figure 1** shows the detailed screening process for eligible participants.

The inclusion criteria were as follows: singleton pregnancy; complete early pregnancy data available; neck circumference, waist circumference, and hip circumference measurements recorded; and available 75 g oral glucose tolerance test (OGTT) at weeks 24–28. The exclusion criteria were comorbid autoimmune disease, chronic hypertension, heart disease, thyroid disorders, Diabetes Mellitus, and severe liver or kidney disease. This study was approved by the ethics committee of the Maternal and Child Health Hospital of Qinhuangdao, and strictly complied with the principles of the Declaration of Helsinki.

### Data collection

All relevant information was collected from medical record system, including maternal age, family history, height, prepregnancy weight and systolic blood pressure, diastolic blood pressure, waist circumference, blood alanine aminotransferase (ALT), aspartate aminotransferase (AST), γ-glutamyl transpeptidase (GGT), uric acid (UA), total cholesterol (TC), triglyceride (TG), high-density lipoprotein cholesterol (HDL-C), low-density lipoprotein cholesterol (LDL-C), glycosylated hemoglobin A1c(HbA1c) and fasting insulin (FINS) in early pregnancy (weeks 8–12 of pregnancy). And the 0 h, 1 h, and 2 h plasma glucose levels in OGTT at mid-pregnancy (weeks 24–28 of gestation) were also collected.

Indices were calculated: BMI = Weight(kg)/Height^2^(m^2^); TyG index =ln [TG (mg/dl)× FPG (mg/dl)/2]; Homeostasis model evaluation insulin resistance index (HOMA-IR) =FPG (mmol/L)×FINS (μU/ml)/22.5; VAI (female)=WC/(36.58 + 1.89 × BMI)× TG/0.81 ×1.52/HDL-C; FLI = [e0.953 × ln(TG) + 0.139 × BMI + 0.718 × ln(GGT) + 0.053 × WC 15.745]/[1 + e0.953 × ln(TG) + 0.139 × BMI + 0.718 × ln(GGT) + 0.053 × WC 15.745] × 100.

### Diagnostic criteria and grouping

GDM diagnostic criteria: The diagnostic criteria of the International Association of Diabetes and Pregnancy Study Groups (IADPSG) (15) were used. Pregnant women who were not diagnosed with Diabetes Mellitus before pregnancy or during early pregnancy underwent 75 g OGTT at 24–28 weeks of gestation. Blood glucose was measured on an empty stomach at 1 h and 2 h after glucose challenge. GDM was diagnosed when the participant had either fasting blood glucose (FPG) ≥5.1 mmol/L, 1 h post-blood glucose(1hPG) ≥10.0 mmol/L, or 2 h post-blood glucose (2hPG) ≥8.5 mmol/L.

The participants were divided into two groups according to their OGTT results: (1) the normal gestational glucose tolerance (NGT) group and (2) the GDM group, in which blood glucose met the aforementioned GDM diagnostic criteria.

### Statistical analysis

SPSS 25.0 statistical software was used for the analyses. Normally distributed quantitative data are expressed as x ± s, nonnormally distributed quantitative data are expressed as M(P25, P75), and the quantitative data are expressed as percentages (%). Data between the NGT group and GDM group were compared using *t* test, χ^2^ test and nonparametric test. In this study, participants were divided into tertiles based on their calculated FLI scores. The lowest tertile (T1) represented the lowest 33.3% of intake, the middle tertile (T2) represented the middle 33.3%, and the highest tertile (T3) represented the highest 33.3%. Chi-square test and analysis of variance were used to compare the data among the three tertiles. Multivariate logistic regression models were employed to explore the independent relationship between FLI and GDM in three different models. In Model 1, age, prepregnancy BMI were adjusted. Model 2 was adjusted for Model 1 adds early pregnancy FPG, early pregnancy HbA1c, LDL-C, and UA. Model 3 was adjusted for Model 2 adds VAI, family history of diabetes. Odds ratios (ORs) and 95% confidence intervals (CIs) were determined. The area under the curve (AUC) of the receiver operating characteristic (ROC) curve was calculated to evaluate the predictive capacity of the FLI for GDM, and the highest Youden index was used to calculate the optimal prediction cutoff point. A difference with *P* < 0.05 was considered to be significant.

## Result

### Comparison of clinical data between the groups

As shown in **Table 1**, there were no significant differences in height, prepregnancy weight, prepregnancy BMI, systolic blood pressure, diastolic blood pressure, early pregnancy waist circumference, HDL-C, TC, ALT, AST, ALT/AST, or family history of diabetes between the two groups (*P*>0.05). In the GDM group, early pregnancy TG, LDL-C, TG/HDL-C, LDL-C/HDL-C, TC/HDL-C, TyG, FLI, UA, and GGT levels were significantly greater than those in the NGT group (*P* < 0.05), and the mid-pregnancy OGTT 0hPG, 1hPG, and 2hPG levels were significantly greater than those in the NGT group (*P* < 0.05).

**Table 1:** Comparison of basic information and biochemical indices between the two groups.

Index	GDM	Non-GDM	*t/χ^2^/Z* value	*P-*value
Number of cases	188	816		
Age (years)	29.17 ± 3.83	27.96 ± 3.64	3.926	0.000
Height(cm)	162.47 ± 4.21	162.97 ± 4.26	-1.454	0.146
Prepregnancy Weight(kg)	56.84 ± 9.80	56.47 ± 9.46	0.482	0.630
BMI(kg/m^2^)	21.53 ± 3.62	21.25 ± 3.39	0.993	0.321
Systolic pressure(mmHg)	116.26 ± 9.91	115.60 ± 10.33	0.795	0.427
Diastolic pressure (mmHg)	70.05 ± 7.59	69.66 ± 8.01	0.603	0.546
WC (cm)	71.62 ± 6.36	71.60 ± 6.09	0.031	0.975
FPG (mmol/L)	4.84 ± 0.44	4.75 ± 0.44	2.270	0.023
TG (mmol/L)	1.72 (1.42,2.15)	1.40 (1.12,1.75)	-7.923	0.000
HDL-C (mmol/L)	1.88 ± 0.41	1.93 ± 0.41	-1.536	0.125
LDL-C (mmol/L)	2.08 ± 0.71	1.88 ± 0.65	3.752	0.000
TC (mmol/L)	4.52 ± 0.79	4.50 ± 0.76	0.352	0.725
UA(μmol/L)	233.94 ± 49.01	204.89 ± 46.99	7.581	0.000
AST (U/L)	16.25 (14.50,19.60)	16.40 (14.20,19.50)	-0.409	0.683
ALT(U/L)	12.00 (9.10,16.68)	11.90 (8.90,17.00)	-0.100	0.921
GGT (U/L)	12.00 (9.00,16.50)	11.00 (8.80,14.58)	-2.234	0.025
ALT/AST	0.74 (0.58,0.93)	0.74 (0.57,1.03)	-0.279	0.780
TyG index	8.83 ± 0.37	8.59 ± 0.35	8.544	0.000
TG/HDL-C	0.96 (0.71,1.23)	0.72 (0.55,0.97)	-6.993	0.000
LDL-C/HDL-C	1.18 ± 0.56	1.03 ± 0.44	3.607	0.000
TC/HDL-C	2.50 ± 0.63	2.41 ± 0.53	2.075	0.038
HOMA-IR	2.69 (1.90,3.79)	1.66 (1.08,2.62)	-8.635	0.000
HbA1c (%)	5.07 ± 0.37	4.89 ± 0.30	6.188	0.000
family history(%)	15 (7.98%)	65 (7.97%)	0.000	0.995
VAI	1.66 (1.18,1.66)	1.28 (0.98,1.69)	-6.684	0.000
FLI	8.47 (5.26,13.41)	6.10 (3.90,10.41)	-5.028	0.000
Pregnancy weeks 24 to 28				
0h Plasma glucose (mmol/L)	5.27 ± 0.42	4.54 ± 0.29	22.487	0.000
1h Plasma glucose (mmol/L)	9.38 ± 1.72	7.21 ± 1.16	16.506	0.000
2h Plasma glucose (mmol/L)	7.94 ± 1.36	6.17 ± 0.96	16.815	0.000

BMI, body mass index; WC, waist circumference; FPG, fasting plasma glucose; TG, triglyceride; TC, total cholesterol; HDL-C, high density lipoprotein cholesterol; LDL-C, low density lipoprotein cholesterol; UA, serum uric acid; ALT, alanine aminotransferase; AST, aspartate aminotransferase; GGT, gamma glutamyl transpeptidase; TyG index, triglycerides/glucose index; HOMA-IR, homeostasis model assessment of insulin resistance; HbA1c, glycosylated hemoglobin A1c; VAI, visceral adipose index; FLI, fatty liver index; GDM, Gestational Diabetes Mellitus.

The total population was divided into three groups according to FLI tertiles. The GDM ratios of the three groups were T1: 11.34%, T2: 19.10%, and T3: 25.75%, indicating that the GDM ratio increased with the FLI tertile (*P* < 0.001). Among these factors, there were differences in age, prepregnancy weight, prepregnancy BMI, systolic blood pressure, early pregnancy waist circumference, TG, TC, HDL-C, LDL-C, UA, AST, ALT, GGT, TG/HDL-C, LDL-C/HDL-C, TC/HDL-C, TyG, HOMA-IR, HbA1c and VAI (*P* < 0.05). There were significant differences in midpregnancy OGTT 0h, 1h, and 2h plasma glucose (*P* < 0.001). There were no significant differences in height, FPG, or family history of diabetes between the different groups (*P*>0.05). See **Table 2**.

**Table 2:** Comparison of general data between the FLI tertiles.

Item	FLI-T1 (n = 335)	FLI-T2 (n = 335)	FLI-T3 (n = 334)	*F/χ2*	*P*
Age (years)	27.33 ± 3.06	28.27 ± 3.74#	28.97 ± 4.07#	17.136	<0.001
Height (cm)	162.99 ± 4.11	163.04 ± 4.25	162.61 ± 4.39	1.034	0.356
Prepregnancy Weight (cm)	50.61 ± 5.59	55.52 ± 6.85#	63.51 ± 10.47#	227.304	<0.001
BMI (kg/m^2^)	19.05 ± 1.91	20.86 ± 2.11#	24.01 ± 3.78#	278.10	<0.001
SDP(mmHg)	115.17 ± 10.32	114.90 ± 9.76	117.11 ± 10.54#	4.646	0.010
DBP(mmHg)	69.24 ± 7.64	69.19 ± 7.96	70.77 ± 8.12#	4.279	0.014
WC(cm)	68.99 ± 4.34	71.62 ± 5.10#	74.21 ± 7.41#	68.651	<0.001
FPG (mmol/L)	4.75 ± 0.45	4.76 ± 0.44	4.80 ± 0.43	1.070	0.343
TG (mmol/L)	1.19 (0.99,1.42)	1.47 (1.21,1.77)#	1.81 (1.45,2.29)#&	268.508	<0.001
HDL-C (mmol/L)	1.97 ± 0.40	1.92 ± 0.40	1.86 ± 0.42#	6.603	0.001
LDL-C (mmol/L)	1.79 ± 0.61	1.98 ± 0.66#	1.99 ± 0.70#	9.299	<0.001
TC (mmol/L)	4.31 ± 0.71	4.55 ± 0.75#	4.66 ± 0.80#	18.688	<0.001
UA(μmol/L)	201.47 ± 47.43	207.63 ± 45.88	221.93 ± 50.50#	16.008	<0.001
AST(U/L)	16.00 (14.00,18.20)	16.20 (14.00,18.90)	17.70 (15.00,22.72)#&	28.355	<0.001
ALT(U/L)	10.40 (8.00,13.90)	11.80 (8.60,16.00)#	14.75 (11.00,24.50)#&	90.587	<0.001
GGT(U/L)	9.20 (8.00, 12.00)	10.80 (8.80,14.00)#	14.90 (11.00,22.80)#&	207.520	<0.001
ALT/AST	0.64 (0.52,0.87)	0.73 (0.59,0.96)#	0.84 (0.66,1.15)#&	74.886	<0.001
TyG	8.41 ± 0.28	8.63 ± 0.31#	8.86 ± 0.37#	167.832	<0.001
TG/HDL-C	0.62 (0.48,0.75)	0.76 (0.60,1.02)#	0.97 (0.74,1.37)#&	216.466	<0.001
LDL-C/HDL-C	0.95 ± 0.41	1.08 ± 0.45#	1.13 ± 0.51#	13.175	<0.001
TC/HDL-C	2.24 ± 0.45	2.44 ± 0.52#	2.60 ± 0.61#	37.933	<0.001
HOMA-IR	1.44 (0.97,2.11)	1.89 (1.16,2.97)#	2.35 (1.55,3.72)#&	93.404	<0.001
HbA1c (%)	4.85 ± 0.30	4.92 ± 0.33#	5.01 ± 0.31#	20.125	<0.001
0h Plasma glucose (mmol/L)	4.56 ± 0.38	4.69 ± 0.45#	4.76 ± 0.44#	15.423	<0.001
1h Plasma glucose (mmol/L)	7.23 ± 1.35	7.62 ± 1.63#	7.99 ± 1.53#	20.894	<0.001
2h Plasma glucose (mmol/L)	6.25 ± 1.06	6.48 ± 1.32#	6.79 ± 1.30#	16.098	<0.001
Family history (%)	21 (6.27%)	31 (9.25%)	28 (8.38%)	2.153	0.341
VAI	1.10 (0.86,1.35)	1.40 (1.05,1.77)#	1.66 (1.25,2.37)#&	173.021	<0.001
FLI	3.49 (2.74,4.07)	6.43 (5.58,7.51)#	14.46(10.96,21.58)#&	891.556	<0.001
GDM (%)	38 (11.34%)	64 (19.10%)#	86 (25.75%)#	22.852	<0.001

#*P* < 0.05 compared with FLI-T1; &*P* < 0.05 compared with FLI-T2.

BMI, body mass index; WC, waist circumference; FPG, fasting plasma glucose; TG, triglyceride; TC, total cholesterol; HDL-C, high density lipoprotein cholesterol; LDL-C, low density lipoprotein cholesterol; UA, serum uric acid; ALT, alanine aminotransferase; AST, aspartate aminotransferase; GGT, gamma glutamyl transpeptidase; TyG index, triglycerides/glucose index; HOMA-IR, homeostasis model assessment of insulin resistance; HbA1c, glycosylated hemoglobin A1c; VAI, visceral adipose index; FLI, fatty liver index; GDM, Gestational Diabetes Mellitus.

Multivariate logistic regression analysis results show that, after correcting for age, prepregnancy BMI, early pregnancy FPG, HbA1c, LDL-C, UA, VAI and the family history of diabetes, GDM risk increased with FLI (OR = 1.881, 95% CI: 1.049–3.374, *P* = 0.034), and the risk of developing GDM in pregnant women in the highest FLI tertile was 1.881 times greater than that in the lowest FLI tertile. See **Table 3**.

**Table 3:** Logistic regression analysis of FLI and GDM.

Group	Odds ratio (95% CI), p value					
	Model 1 (n=335)		Model 2 (n=335)		Model 3 (n=334)	
T1	1		1		1	
T2	1.979 (1.264–3.100)	0.003	1.757 (1.096–2.818)	0.019	1.524 (0.940–2.471)	0.088
T3	3.528 (2.143–5.806)	<0.001	2.642 (1.560–4.475)	<0.001	1.881 (1.049–3.374)	0.034

Model 1: corrected age, prepregnancy BMI; Model 2: Model 1+ early pregnancy fasting blood glucose, early pregnancy glycated hemoglobin, LDL-C, and UA; Model 3: Model 2+VAI, family history of diabetes.

**Figure 2:**
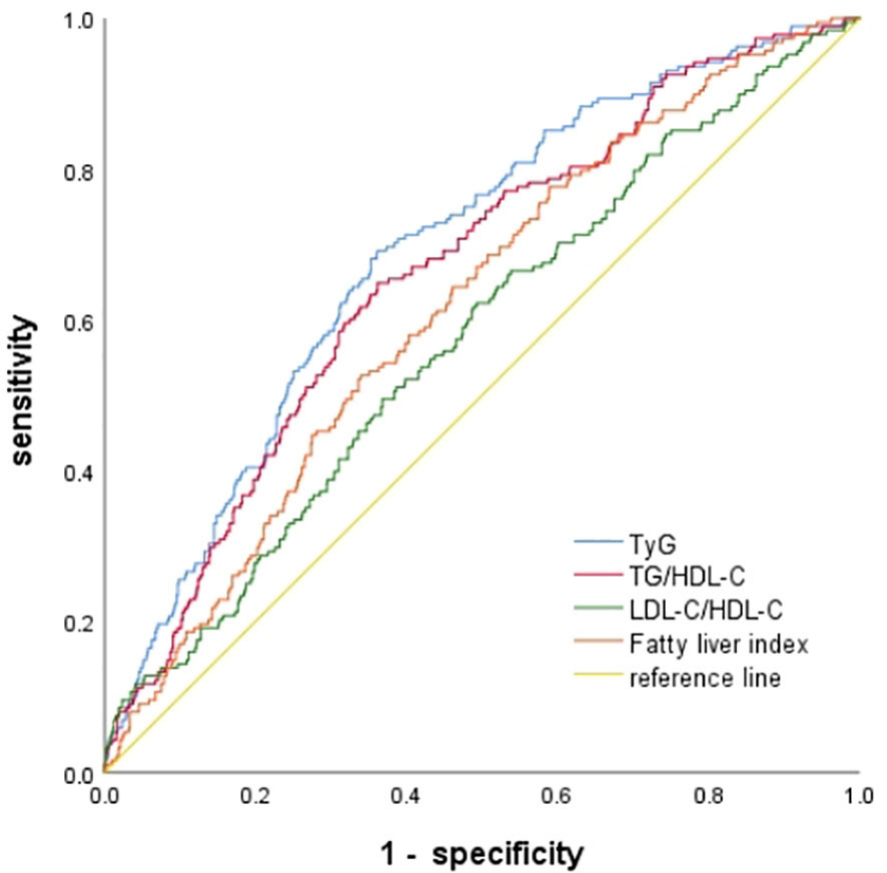
ROC curve of each indicator predicting the risk of GDM.

ROC curves were used to analyze and compare the predictive value of TyG, TG/HDL-C, LDL-C/HDL-C and the FLI for GDM. The cut-off point of FLI for predicting the risk of GDM was 5.108. Compared with other three indicators, FLI has better sensitivity(77.7%), but the specificity was slightly lower(41.1%). See **Table 4**; **Figure 2**.

**Table 4:** Predictive value of TG/HDL-C, LDL-C/HDL-C, TyG, and FLI for GDM.

Parameter	Cut point	AUC	95% CI	Sensitivity	Specificity	Positive predictive value	Negative predictive value	Youden index	*P* value
TyG	8.683	0.692	0.652–0.733	69.1	63.8	30.6	90.0	0.329	<0.001
TG/HDL-C	0.831	0.663	0.622–0.705	64.9	63.8	29.3	88.8	0.287	<0.001
LDL-C/HDL-C	0.977	0.576	0.531–0.621	62.2	50.8	22.5	85.4	0.129	0.001
FLI	5.108	0.617	0.575–0.660	77.7	41.1	23.3	88.9	0.188	<0.001

TG, triglyceride; HDL-C, high density lipoprotein cholesterol; LDL-C, low density lipoprotein cholesterol; TyG index, triglycerides/glucose index; FLI, fatty liver index.

## Discussion

MAFLD is an important risk factor for the occurrence and progression of GDM, is associated with severe adverse maternal and infant outcomes, and has potential long-term effects on mothers and their descendants (16, 17). The FLI is a noninvasive surrogate marker for evaluating hepatic steatosis and has attracted increasing attention because it is convenient, inexpensive, and easy to measure. The FLI can be a predictor or risk factor for many metabolic and nonmetabolic diseases (18). The clinical significance of the FLI in pregnant women has not been well determined. Our study examined the possible correlation between FLI-assessed hepatic steatosis and GDM.

A prospective South Korean study evaluated liver ultrasound and FLI in early pregnant women and revealed that the risk of developing GDM is significantly increased in NAFLD patients and is positively correlated with steatosis severity (16). A similar study in Canada revealed that hepatic steatosis in early pregnancy is associated with impaired fasting blood glucose and glucose tolerance at 24–28 weeks of gestation or GDM (19). A meta-analysis revealed that the prevalence of GDM is 26.0% in women with NAFLD. Compared with non-NAFLD women, the probability of developing GDM in NAFLD pregnant women is more than 2.9 times greater (20).

MAFLD identification is based on the identification of hepatic steatosis (through liver biopsy, imaging, or blood-based biomarkers). Liver biopsy is the gold standard for diagnosing NAFLD; however, it is unsuitable for pregnant women. NAFLD diagnosis is mainly based on ultrasonography. However, liver ultrasound is a relatively weak method for diagnosing NAFLD, particularly when hepatic steatosis is mild (21). In recent years, an increasing number of studies have shown that FLI can replace ultrasound in determining the severity of hepatic steatosis. The FLI was first proposed by Bedogni et al. in 2006 (8), and this marker is calculated on the basis of BMI, waist circumference, triglycerides, and γ-glutamyl transpeptidase. This multivariate model includes biomarkers and can accurately evaluate the presence of fatty liver disease; it is one of the most widely used and effective markers in the world (22). Therefore, the FLI has been recommended as the primary diagnostic tool for large-scale screening research in the guidelines for NAFLD by the European Association for the Study of the Liver (EASL), the European Association for the Study of Diabetes (EASD), and the European Association for the Study of Obesity (EASO) (23). A study by Tina Linder (24) revealed that the hepatic steatosis index is associated with impaired glucose regulation and could provide a useful tool for early risk assessment of impaired glucose metabolism. A European study (25) revealed that the FLI is closely associated with GDM, especially with insulin resistance and inflammation. Our findings were consistent with those of the aforementioned studies, as our results showed that the FLI value in the GDM group was greater than that in the NGT group, and the difference was significant (P<0.001). FLI tertiles were used for grouping, and the GDM detection rate gradually increased with increasing FLI, with values of 11.34% for T1, 19.10% for T2, and 25.75% for T3 (P<0.001). These findings indicate that early pregnancy FLI is an independent risk factor for GDM.

The mechanism by which a high FLI is associated with GDM occurrence remains unclear. However, many previous studies have shown that the FLI is closely associated with insulin resistance and T2DM (26). The results of an Italian study revealed that the FLI is not only a marker of liver fat content (through MRS measurement) but also linearly correlated with insulin resistance (27). The study of Bozkurt (25) evaluated the relationship between women with previous GDM and the risk of postpartum fatty liver disease (evaluated via the FLI). Sixty-eight women with a previous diagnosis of GDM and 29 healthy controls were included 3–6 months after delivery and underwent specific metabolic evaluation, with a particular focus on the relationship between insulin resistance and proinflammatory factors. The results showed that the postpartum FLI is closely associated with previous GDM, especially insulin resistance, and inflammation. In addition, a prospective study on the relationship between the postpartum early FLI and the development of type 2 Diabetes Mellitus during a 10-year follow-up was conducted. Interestingly, FLI-assessed fatty liver disease is associated with a possible increase in diabetes during the 10-year follow-up (25). A European study (24) analyzed the metabolic characteristics of 109 women at 16 weeks of gestation and divided them into low-risk (G1), intermediate-risk (G2), and high-risk (G3) groups according to the FLI. β-cell function and GDM status were evaluated at week 26 of gestation. The early pregnancy FLI is closely associated with impaired glucose metabolism regulation pathophysiological characteristics, such as impaired insulin action and β-cell dysfunction. This association could explain why the risk of developing GDM in pregnant women with a high FLI is increased (24). Our study revealed that when FLI tertiles were used for grouping, HOMA–IR gradually increased with FLI in the T1–T3 groups, and the differences were significant (P<0.001). Moreover, surrogate markers for insulin resistance, such as TyG, TG/HDL-C, LDL-C/HDL-C, TC/HDL-C, and ALT/AST, gradually increased (P<0.001). These findings indicate that the FLI is associated with insulin resistance.

Owing to differences in anthropometric measurements, there may be differences in FLI distribution between different ethnicities. In this study, there was substantial variability in the threshold value of the FLI. In the African Kenyan cohort, the cutoff point for FLI prediction of NAFLD was 6.12 (28). In Western China, the optimal cutoff point for NAFLD diagnosis is 30.42 (29). In a study of 12,794 Uyghur adults in China, the optimal cutoff point for MAFLD diagnosis in both sexes was 45 (30). Similarly, there are sex differences in the FLI cutoff point. A Taiwanese study (31)revealed that when the FLI cutoff point for fatty liver disease identification was set to 20 in males, the sensitivity was 80.3%, and the specificity was 66.9%. In female participants, when the FLI cutoff point was set to 10, the sensitivity was 76.1%, and the specificity was 65.5%. However, when the FLI cutoff point was set to 60 for fatty liver disease prediction, the sensitivity decreased to 28.4% and 11.5% in males and females, respectively (31). That study concluded that the FLI cutoff point for diagnosing steatosis can be set to 10 in females and 20 in males to increase the feasibility of undergoing AU in patients. Another Taiwanese study (32) also revealed that the FLI can accurately identify fatty liver disease in Taiwanese populations, but the cutoff point is lower than that in Western populations. Moreover, the cutoff point for females is lower than that for males. Another previous study demonstrated similar findings (33). In a cohort of 1,976 Asian participants, the FLI cutoff point for NAFLD prediction was 10.927 in females and 34.522 in males. Similarly, in two US cohorts, when participants were stratified by waist circumference and BMI, the FLI for fatty liver disease prediction was greater in males (48.57 and 61.47) than in females (41.93 and 51.65) (34). Other studies (35) demonstrated similar findings. When the FLI cutoff point was set to 60, the sensitivity also decreased (28.4% in males and 11.5% in females). Conversely, in an Iranian cohort, the FLI cutoff point for fatty liver disease in males was lower than that in females (46.9 and 53.8, respectively) (36). An Italian study revealed that the FLI is the best noninvasive predictor of fatty liver disease. The FLI cutoff point for MAFLD detection in females was 50% lower than that in males. Gender differences are considered to be due to greater MAFLD prevalence and severity in males than in females, whereas the incidence of MAFLD is greater in postmenopausal women. This finding shows that even though biomarkers may indicate that a subject is healthy, customized MAFLD screening and prevention plans should be formulated for different sexes. Therefore, they emphasized the necessity of revising FLI cutoff points (37).

The mean FLI of our pregnant population was significantly lower than that of the European study population, possibly because of significant differences in the mean BMI and waist circumference between our population and the pregnant European population. A European pregnant population study (24) revealed that the BMIs for FLI low-risk (G1), intermediate-risk (G2), and high-risk (G3) patients were 21.9 ± 2.0, 26.1 ± 2.5, and 35.5 ± 4.3, respectively, and the waist circumferences were 85.2 ± 5.5, 97.4 ± 5.3, and 113.5 ± 10.9 cm, respectively. However, in our study, the BMIs of the FLI tertiles were 19.05 ± 1.91, 20.86 ± 2.91, and 24.01 ± 3.78, respectively, and the waist circumferences were 68.99 ± 4.34, 71.62 ± 5.10, and 74.21 ± 7.41 cm, respectively. These values are all significantly lower than those reported in the European pregnant population, and waist circumference, BMI, and the main markers for calculating the FLI were also significantly different.

Although FLI showed higher sensitivity than other indices, its specificity was relatively low (41.1%). The reason for the low specificity might be that during the early stage of pregnancy, the mother’s body undergoes physiological lipid metabolism changes (such as a slight increase in triglycerides) to adapt to the fetal development. These changes are not clearly distinguishable from abnormal metabolic conditions in pathology, making it difficult to precisely differentiate between physiological metabolic fluctuations and the pathological tendency of developing gestational diabetes. When applying in clinical settings, it is necessary to comprehensively evaluate information such as the pregnant woman’s pre-pregnancy medical history (such as fatty liver, family history of diabetes) and lifestyle (such as a high-fat diet, sedentary lifestyle), to avoid drawing conclusions solely based on the index values.FLI can be used as a screening tool, an auxiliary means for risk stratification, and it should be used in combination with other indicators.

This study has several limitations. First, this single-center cross-sectional retrospective study does not represent the entire population. Second, we did not validate the severity of hepatic steatosis when performing liver ultrasound. If liver ultrasound can be combined with FLI, this will facilitate the identification of FLI cutoff points and increase the reliability of the study results. Finally, observations and analyses were conducted in this retrospective study. However, a causal relationship between FLI and GDM risk was not established. It should be clarified that FLI reflects metabolic phenotypes related to liver fat, rather than confirmed MAFLD, and only correlations were proposed. Therefore, our study results should be interpreted with caution and need to be further demonstrated in future studies. Subsequently, multicenter validation studies are warranted to fully validate the clinical utility of this approach, thereby facilitating its translational implementation and widespread clinical application.

GDM is usually diagnosed at 24–28 weeks of gestation. Therefore, the intervention window is very short, and determining the potential risk factors for GDM is important for risk stratification and management. Examining early biomarkers of GDM and further understanding their roles in GDM is important. Early pregnancy FLI is associated with GDM, as it may contribute to increased insulin resistance and has been identified as an independent risk factor for GDM. The FLI is simple, economical, and easy to calculate, and its level predicts GDM risk. Attention should be given to high FLI populations, and aggressive follow-up and early intervention should be conducted to prevent GDM. Future researchers should validate our study results in other populations, and employing simple NAFLD identification methods may help in the early identification of high-risk mothers.

## Data Availability

The raw data supporting the conclusions of this article will be made available by the authors, without undue reservation.
